# Case Report: A Rare Case of Lupus Nephritis Associated With Mantle Cell Lymphoma

**DOI:** 10.3389/fmed.2021.759279

**Published:** 2021-11-29

**Authors:** Daorina Bao, Ying Tan, Xiaojuan Yu, Bingjie Wang, Hui Wang, Rong Xu, Fude Zhou, Minghui Zhao

**Affiliations:** ^1^Renal Division, Department of Medicine, Peking University First Hospital, Beijing, China; ^2^Institute of Nephrology, Peking University, Beijing, China; ^3^Key Laboratory of Renal Disease, Ministry of Health of China, Beijing, China; ^4^Key Laboratory of Chronic Kidney Disease Prevention and Treatment, Ministry of Education of China, Beijing, China; ^5^Research Units of Diagnosis and Treatment of Immune-Mediated Kidney Diseases, Chinese Academy of Medical Sciences, Beijing, China; ^6^Department of Hematology, Peking University First Hospital, Beijing, China; ^7^Laboratory of Electron Microscopy, Pathological Center, Peking University First Hospital, Beijing, China; ^8^Peking-Tsinghua Center for Life Sciences, Beijing, China

**Keywords:** lupus nephritis, mantle cell lymphoma, secondary SLE, non-Hodgkin lymphoma, acute kidney injury

## Abstract

In this research, we described a very rare case of secondary lupus nephritis associated with B-cell lymphoma. An 84-year-old man was hospitalized at our institute for lower extremity edema persisting for over 2 months. He was diagnosed with systemic lupus erythematosus based on clinical and laboratory criteria, which showed impaired renal function and nephrotic syndrome with predominant hematuria. Renal biopsy showed IV+V lupus nephritis with highly infiltrated lymphoid cells in the kidney. Secondary lupus nephritis was suspected based on the possible pathogenesis of glomerular injury due to mantle cell lymphoma. Low-dose dexamethasone, rituximab, and lenalidomide were immediately started on the patient, and his renal function was improved after the first cycle of chemotherapy.

## Background

Lupus nephritis is a form of glomerulonephritis that constitutes one of the most severe organ manifestations of the autoimmune disease, systemic lupus erythematosus (SLE). In many cases, lupus nephritis is the presenting manifestation that results in the diagnosis of SLE (1). Previous literature indicated that lupus-like syndrome could be caused by infections, hematological malignancies, solid tumors, and so forth (2). However, renal involvement presented as lupus nephritis is rare (3–7). In this research, we describe a rare case of mixed proliferative and membranous lupus nephritis, secondary to mantle cell lymphoma, and partially recovered after chemotherapy.

## Case Presentation

An 84-year-old Chinese man was presented to our department with both lower extremity edema for over 2 months. Initial laboratory investigations demonstrated an elevated serum creatinine of 1.64 mg/dl (normal range: 0.50–1.50 mg/dl; 144.6 μmol/L, corresponding to estimated glomerular filtration rate of 37.95 ml/min/1.73 m^2^ as calculated by the CKD-EPI equation) and decreased albumin of 22.9 g/L (normal range: 40–55 g/L), with proteinuria of 24 g/day (total volume 850 ml). Urinary microscopic examination showed microscopic hematuria (80–100 cells/HPF) and pyuria (full-field of view). Ultrasound examination of the kidneys revealed normal-sized kidneys (left 11.8 cm; right 11.7 cm). Blood leucocyte count was 7,100/μl, hemoglobin was 13.2 g/dl and platelet count was 153,000/μl. The antinuclear antibody (ANA) was positive (1:1,000 titer, homogenous) and the anti-double stranded DNA (anti-dsDNA) antibody was positive with the titer of 1:10. Complement 3 (C3) and 4 (C4) were both reduced to 0.423 g/L (normal range: 0.60–1.50 g/L) and 0.027 g/L (normal range: 0.12– 0.36 g/L), respectively. Anti-glomerular basement membrane (anti-GBM) antibody, anti-neutrophil cytoplasmic antibody (ANCA), Coombs' test, antiphospholipid antibody, serum cryoglobulins, serum and urine immunofixation electrophoresis, and anti-PLA2R antibody were all negative.

The patient had mantle cell lymphoma (MCL) 11 months ago, which was diagnosed through a biopsy of both bowel polyp and axillary lymph nodes and classified as Stage III Group A with low-intermediate risk (lymph nodes and gastrointestinal involvement). There was no further chemical treatment for lymphoma because of its indolence and low risk. Besides, he had a series of metabolic disorders, including diabetes, hypertension, and coronary heart disease, with satisfying controlment and no diabetic retinopathy. He was also diagnosed with myasthenia gravis (MG) IIb type (Ossermann Classification) for 5 years. At the time of diagnosis, the patient was on acetylcholinesterase inhibitor for his MG, which significantly improved his muscle weakness. He was treated with tacrolimus at 0.5 mg per day regularly with remission. The blood drug level of FK-506 was 0 ng/ml.

Upon admission, physical examination revealed blood pressure of 160/90 mmHg, a pulse of 99/min, a temperature of 36.3°C, and a respiratory rate of 18/min. Physical examination showed an obvious vicia-sized lymph node at the right axilla, shifting dullness, and severe edema of both lower limbs.

Bone marrow biopsy and (18)F-FDG Positron Emission Tomography/Computed Tomography (PET/CT) were performed to evaluate lymph nodes and organ involvement. Abnormal clones of B cells accounted for 5.6% of bone marrow cells as determined by bone marrow flow cytometry. On fluorescent *in situ* hybridization (FISH), 43 IGH/CCND1 fusion signals can be seen in 200 interphase cells. (18)F-FDG PET/CT revealed systemic lymphadenopathy without extranodal involvement.

**Figure 1:**
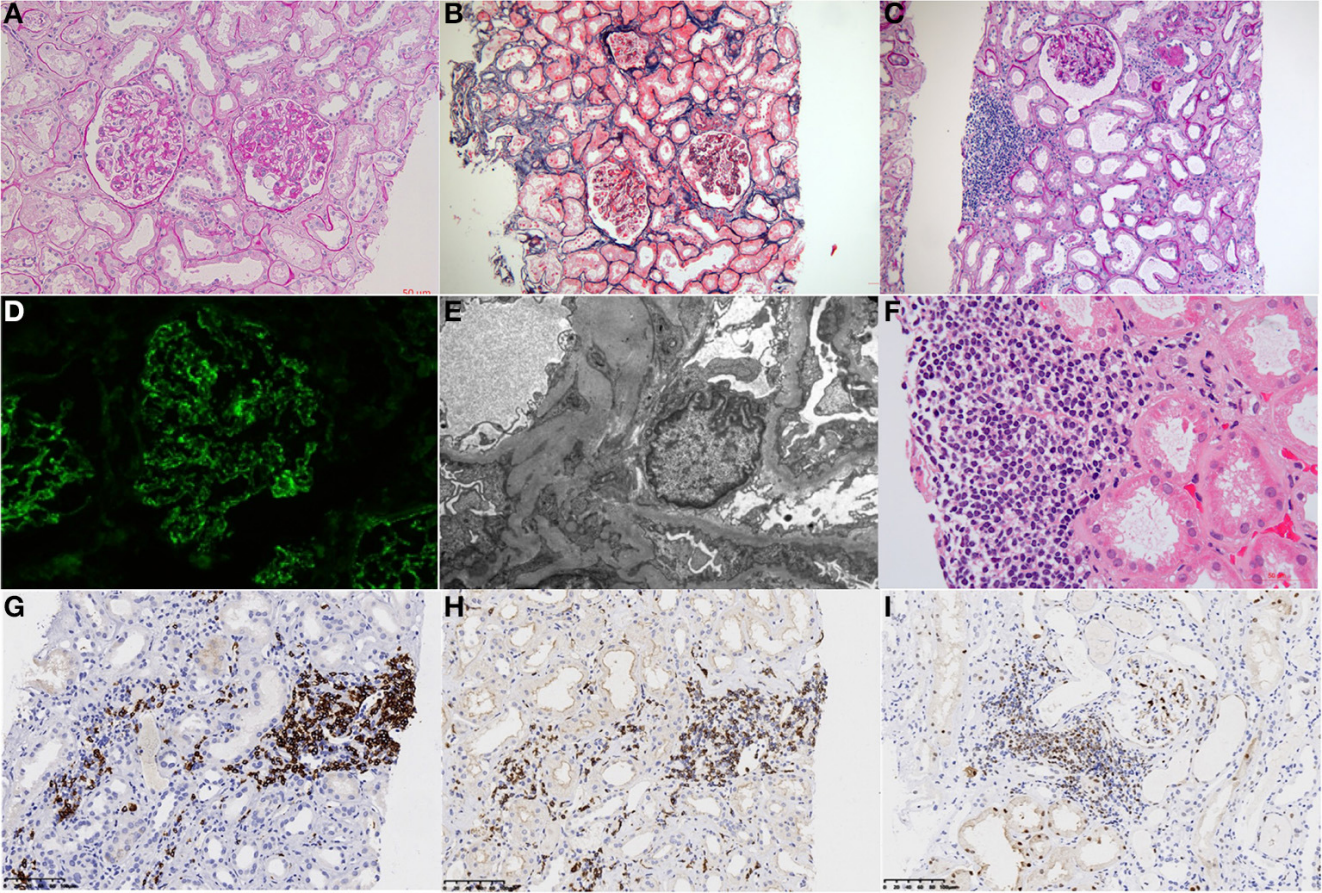
Histological findings on renal biopsy. **(A)** Light microscopy study of the renal biopsy specimen revealed segmental endocapillary proliferation with neutrophil infiltration. (PAS, ×400). **(B)** The tubules displayed acute injury with epithelial simplification and small focal atrophy (Masson trichrome staining, ×200). **(C)** Renal interstitial was infiltrated by focal lymphocytes (×100). **(D)** Immunofluorescence analysis revealed positive granular staining of IgG in the mesangium and capillary wall. **(E)** Electron microscopy showing electron-dense deposits in the sub-epithelial and segmental mesangial. **(F)** Interstitial infiltration of a nodular mass of medium-sized lymphoid cells with irregular nuclei. (PAS, ×400). **(G–I)** Immunohistochemical analysis revealed lymphoblasts were strongly positive for CD 20 **(G)**, CD5 **(H)**, and Cyclin D1 **(I)** (Panels were indicated from left to right with letters **A–I**).

To clarify the pathological changes of his kidney histology, a renal biopsy was performed. Direct immunofluorescence showed full-house staining along mesangium and capillary loops [IgG (3±), IgA (±), IgM (1±), C3 (2±), C1q (1±), fibrinogen (2±), albumin (–), kappa (2±), lambda (3±), IgG1 (1±), IgG2 (1±), IgG3 (1±), and IgG4 (–)]. Light microscopic examination showed that 5/28 glomeruli were ischemic and sclerotic and 1/28 were segmentally sclerotic. The rest of the glomeruli showed mild proliferation of mesangial cells and stroma, accompanied by segmental endothelial cell proliferation and infiltration of neutrophils. The glomerular basement membrane thickened diffusely, with the formation of segmental spikes. The tubules displayed acute injury with epithelial simplification and small focal atrophy. There were scattered proteinaceous and RBC casts. There was intimal fibrous proliferation and sclerosis of small arteries. Congo red stain for amyloid was negative. Renal interstitium was infiltrated by many lymphoma cells with variable sizes, large-oval or irregular nuclei, delicate chromatin pattern, high nuclear-cytoplasm ratio, and prominent nucleoli. Immunohistochemistry showed CD20 (2+), CD3 (1+), CD5 (2+), CyclinD1 (1+), SOX11 (1+), BCL2 (2+), Ki-67 (5%), which confirmed infiltration of mantle cell lymphoma cells in the renal interstitium. Electron microscopy showed that the basement membrane was thickened and diffusely accompanied by sub-epithelial and segmental mesangial electron-dense deposits. Severe foot process effacement was also discovered (Figure 1).

**Figure 2:**
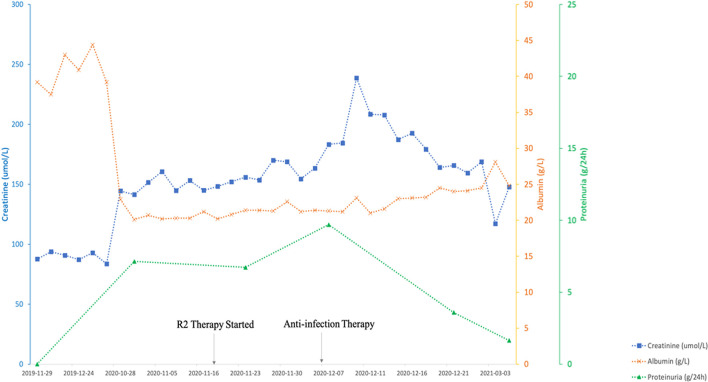
The clinical course of the present case. R2 Therapy indicates Rituximab and lenalidomide.

Chemotherapy treatment with rituximab at 375 mg/m^2^ body surface area (BSA), lenalidomide 25 mg/day for 21 consecutive days, and 10 mg dexamethasone was initiated considering the advanced age and multiple combined disease. The patient developed hospital-acquired pneumonia during the chemotherapy and the renal function rapidly deteriorated as serum creatinine elevated from 1.76 mg/dl (156 μmol/L) to a peak of 2.69 mg/dl (238 μmol/L). After the successful anti-infection therapy was performed, his renal function was improved and the serum creatinine fell back to baseline at 1.32 mg/dl (117 μmol/L). The patient's ANA was 1:100, anti-dsDNA levels negative, and C3 and C4 immediately returned to normal level after just one round of chemotherapy (Figure 2).

## Discussion

Systematic lupus erythematosus (SLE) is an autoimmune disease characterized by multisystem organ involvement, heterogeneity of clinical features, and a variety of degrees of severity, of which lupus nephritis is constituted with one of the most common causes of morbidity and mortality. However, previous studies showed that SLE could be secondary to infections, hematological malignancies, solid tumors, drugs, and so on (2), with renal involvement (3–7).

Herein, we present a case of an 84-year-old man with a history of mantle cell lymphoma for 11 months and was also diagnosed as lupus nephritis confirmed by renal biopsy. The main differential diagnoses considered in our patient were whether the described associated glomerular disease is the paraneoplastic syndrome of lymphoma or that of concurrent primary lupus nephritis. However, new-onset SLE is uncommon in elderly male patients (8), and this patient notably has infiltration of mantle cell lymphoma cells in the renal interstitium and bone marrow. Besides, our patient achieved rapid remission of the glomerular disease and immune system remission after the first chemotherapy for MCL. The paraneoplastic nature of renal lesions made us consider that lupus nephritis was secondary to MCL.

Mantle cell lymphoma (MCL) is a unique type of B-cell non-Hodgkin lymphoma (NHL), which is considered aggressive. Several case reports have been published on the glomerular involvement of MCL. Some of the histopathological findings include minimal change disease (MCD) (9), membranoproliferative glomerulonephritis (MPGN) (10), proliferative glomerulonephritis (11), and ANCA-associated pauci-immune crescentic glomerulonephritis (12).

Lupus nephritis secondary to MCL is indeed rare. As far as we know, the first and the only case of lupus nephritis associated with MCL was reported in 2018 (5). No study, to date, has reported a specific mechanism and relationship between SLE and MCL. Wang et al. (13) performed a nation-wide observational study in Taiwan, evaluating if there was a bidirectional relationship between SLE and NHL. They found that the patients with NHL had a higher risk of SLE. Morth et al. (14) illustrated that the most common specific autoimmune diseases, which were categorized as primarily B-cell mediated, were rheumatoid arthritis, SLE, and primary Sjögren's syndrome in patients with diffuse large B-cell lymphoma. The suspected pathogenesis shared by some autoimmune diseases and NHL might include similar genetic risk factors or trigger factors (13). A previous study concluded that both T cells and B cells have important roles in SLE pathogenesis. Especially, B cells produce the hallmark autoantibodies like anti-DNA antibodies and antinuclear antibodies (1). T follicular helper cells, which were recognized as a novel subpopulation of helper T cells (7), activate germinal center B cells to produce autoantibodies. The expansion of lymphoma cells has efficient ways to influence the immune system toward dysregulation or chronic stimulation, which may foster SLE development in individuals who are also prone to autoimmune diseases (15).

## Conclusion

In summary, we reported an old man who presented with nephrotic syndrome and acute kidney disease attributable to lupus nephritis. Renal biopsy showed mixed proliferative and membranous lupus nephritis with concomitant interstitial infiltration from lymphoid cells. The patient was considered to be a case of lupus nephritis secondary to mantle cell lymphoma. However, the underlying pathogenesis in lymphoma-associated lupus nephritis still needs to be addressed.

## Data Availability Statement

The original contributions presented in the study are included in the article/supplementary material, further inquiries can be directed to the corresponding author/s.

## Ethics Statement

The studies involving human participants were reviewed and approved by Peking University First Hospital, approval number: 2017[1333]. The patients/participants provided their written informed consent to participate in this study. Written informed consent was obtained from the individual(s) for the publication of any potentially identifiable images or data included in this article.

## Author Contributions

DB, YT, and BW analyzed and interpreted the patient data and were major contributors in writing the manuscript. XY and HW performed interpretation of pathological data. RX, FZ, and MZ performed interpretation of the clinical data and substantively revised it. All authors contributed to the article and approved the submitted version.

## Funding

This study was supported by Beijing Natural Science Foundation (No. 7192207) and CAMS Innovation Fund for Medical Sciences (2019-I2M-5-046).

## Conflict of Interest

The authors declare that the research was conducted in the absence of any commercial or financial relationships that could be construed as a potential conflict of interest.

## Publisher's Note

All claims expressed in this article are solely those of the authors and do not necessarily represent those of their affiliated organizations, or those of the publisher, the editors and the reviewers. Any product that may be evaluated in this article, or claim that may be made by its manufacturer, is not guaranteed or endorsed by the publisher.
